# Mutualistic Interactions between Dinoflagellates and Pigmented Bacteria Mitigate Environmental Stress

**DOI:** 10.1128/spectrum.02464-22

**Published:** 2023-01-18

**Authors:** Toshiyuki Takagi, Kako Aoyama, Keisuke Motone, Shunsuke Aburaya, Hideyuki Yamashiro, Natsuko Miura, Koji Inoue

**Affiliations:** a Atmosphere and Ocean Research Institute, The University of Tokyo, Kashiwa, Japan; b Graduate School of Frontier Sciences, The University of Tokyo, Kashiwa, Japan; c Paul G. Allen School of Computer Science and Engineering, University of Washington, Seattle, Washington, USA; d Graduate School of Agriculture, Osaka Metropolitan University, Sakai, Japan; e Division of Metabolomics, Medical Institute of Bioregulation, Kyushu University, Fukuoka, Japan; f Tropical Biosphere Research Center, Sesoko Station, University of the Ryukyus, Motobu, Japan; Swansea University

**Keywords:** dinoflagellate, Symbiodiniaceae, coral holobiont, pigmented bacteria, microbiome manipulation, carotenoid

## Abstract

Scleractinian corals form symbiotic relationships with a variety of microorganisms, including endosymbiotic dinoflagellates of the family Symbiodiniaceae, and with bacteria, which are collectively termed coral holobionts. Interactions between hosts and their symbionts are critical to the physiological status of corals. Coral-microorganism interactions have been studied extensively, but dinoflagellate-bacterial interactions remain largely unexplored. Here, we developed a microbiome manipulation method employing KAS-antibiotic treatment (kanamycin, ampicillin, and streptomycin) to favor pigmented bacteria residing on cultured *Cladocopium* and *Durusdinium*, major endosymbionts of corals, and isolated several carotenoid-producing bacteria from cell surfaces of the microalgae. Following KAS-antibiotic treatment of *Cladocopium* sp. strain NIES-4077, pigmented bacteria increased 8-fold based on colony-forming assays from the parental strain, and 100% of bacterial sequences retrieved through 16S rRNA amplicon sequencing were affiliated with the genus *Maribacter*. Microbiome manipulation enabled host microalgae to maintain higher maximum quantum yield of photosystem II (variable fluorescence divided by maximum fluorescence [*F_v_*/*F_m_*]) under light-stress conditions, compared to the parental strain. Furthermore, by combining culture-dependent and -independent techniques, we demonstrated that species of the family Symbiodiniaceae and pigmented bacteria form strong interactions. Dinoflagellates protected bacteria from antibiotics, while pigmented bacteria protected microalgal cells from light stress via carotenoid production. Here, we describe for the first time a symbiotic relationship in which dinoflagellates and bacteria mutually reduce environmental stress. Investigations of microalgal-bacterial interactions further document bacterial contributions to coral holobionts and may facilitate development of novel techniques for microbiome-mediated coral reef conservation.

**IMPORTANCE** Coral reefs cover less than 0.1% of the ocean floor, but about 25% of all marine species depend on coral reefs at some point in their life cycles. However, rising ocean temperatures associated with global climate change are a serious threat to coral reefs, causing dysfunction of the photosynthetic apparatus of endosymbiotic microalgae of corals, and overproducing reactive oxygen species harmful to corals. We manipulated the microbiome using an antibiotic treatment to favor pigmented bacteria, enabling their symbiotic microalgal partners to maintain higher photosynthetic function under insolation stress. Furthermore, we investigated mechanisms underlying microalgal-bacterial interactions, describing for the first time a symbiotic relationship in which the two symbionts mutually reduce environmental stress. Our findings extend current insights about microalgal-bacterial interactions, enabling better understanding of bacterial contributions to coral holobionts under stressful conditions and offering hope of reducing the adverse impacts of global warming on coral reefs.

## INTRODUCTION

Coral reefs cover less than 0.1% of the ocean floor, but about 25% of all marine species depend on them at some point during their life cycles; thus, reefs are crucially important ecosystems for conservation of marine biodiversity, including corals, fish, shellfish, and many other organisms (1, 2). However, various species of coral are threatened with extinction due to increased anthropogenic disturbances, including global warming (3). Elevated ocean temperatures associated with global climate change are a serious threat to coral reefs and are linked to increasingly frequent coral diseases and mass coral bleaching (4–6).

Coral reef ecosystems are based on symbiotic relationships between the host coral animals and unicellular, photosynthetic dinoflagellates belonging to the family Symbiodiniaceae (7, 8). Corals and their associated microorganisms, including endosymbiotic dinoflagellates, fungi, and bacteria, comprise coral holobionts (9). Among these, endosymbiotic dinoflagellates of the family Symbiodiniaceae provide photosynthetic products to their coral hosts, which, in turn, supply carbon dioxide and inorganic nutrients to these microalgal endosymbionts (10); therefore, these mutualistic relationships are essential. However, long-term stress from higher ocean temperatures and excess insolation result in overproduction of reactive oxygen species (ROS) from symbiotic microalgae, inducing a breakdown of symbiosis, i.e., coral bleaching (11). Unless affected corals reacquire their microalgal partners from the environment, prolonged bleaching leads to coral mortality, resulting in adverse impacts on the ecosystem services provided by coral reefs (12). Bleaching susceptibility varies depending on host responses, including upregulation of antioxidant enzymes, pigment biosynthesis, and changes in the bacterial community (13–15). Furthermore, recent studies on coral holobionts suggest that probiotic bacteria improve coral stress tolerance and mitigate bleaching impacts (16–19).

Microalgal-bacterial relationships also have a significant impact on the health of coral holobionts. Various mutualistic relationships have been reported in microalgal-bacterial interactions (20–25). Microalgae provide fixed organic carbon compounds to bacteria (20), which in turn, supply their microalgal partners with vitamin B_12_ (20–22), plant hormones for promoting host growth (23), and increased levels of bioavailable iron (24). Recently, we demonstrated that the marine Flavobacteriaceae sp. strain GF1, which is most closely related to the genus *Muricauda*, protects microalgal symbionts from environmental stress and inhibits ROS generation through zeaxanthin production (25). The genus *Muricauda* is reportedly one of the core members of bacterial communities in Symbiodiniaceae cultures (26, 27). These carotenoid-producing bacteria, symbiotic with Symbiodiniaceae cells, may help to mitigate mass bleaching events.

Here, we report development of a microbiome manipulation method using antibiotic treatment to favor pigmented bacteria in the presence of *Cladocopium* and *Durusdinium*, major dinoflagellate taxa symbiotic with corals (28), and isolated several carotenoid-producing bacteria from these microalgae. Compared to parent microalgal strains, microbiome manipulation successfully improved light-stress tolerance in cultured Symbiodiniaceae. Furthermore, by combining culture-dependent and -independent techniques, we investigated the mechanisms underlying dinoflagellate-pigmented bacteria interactions and described here, for the first time, a symbiotic relationship in which symbiotic partners mutually reduce environmental stress. Our findings extend understanding of microalgal-bacterial interactions and bacterial functions which promote coral holobiont health under stressful conditions. Such knowledge may help to reduce the adverse impacts of global warming on coral reefs through microbiome manipulation.

## RESULTS

### Antibiotic treatment favors pigmented bacteria in Symbiodiniaceae cultures.

In our previous study, an antibiotic mixture treatment in F/2 agar medium (50 μg/mL kanamycin, 100 μg/mL ampicillin, and 50 μg/mL streptomycin [KAS-antibiotics]) allowed orange-pigmented Flavobacteriaceae sp. GF1, which produces zeaxanthin, to become dominant in a Symbiodiniaceae culture (25). To confirm the effects of the antibiotic mixture on the bacterial community, we performed the antibiotic treatment in two Symbiodiniaceae cultures (*Cladocopium* sp. NIES-4077 and Cladocopium goreaui CCMP2466) (29). These established cultures were named Abx-4077 and Abx-2466, respectively. Furthermore, to assess whether the observed associations of pigmented bacteria and Symbiodiniaceae were artifacts of extended laboratory culture, we freshly isolated Symbiodiniaceae cells from the coral, Galaxea fascicularis, with F/2 agar medium containing KAS-antibiotics. This culture was named *Durusdinium* sp. SGF. After recovering cultivation with F/2 liquid medium without antibiotic, we performed colony-forming assays of bacteria on marine agar plates.

**FIG 1:**
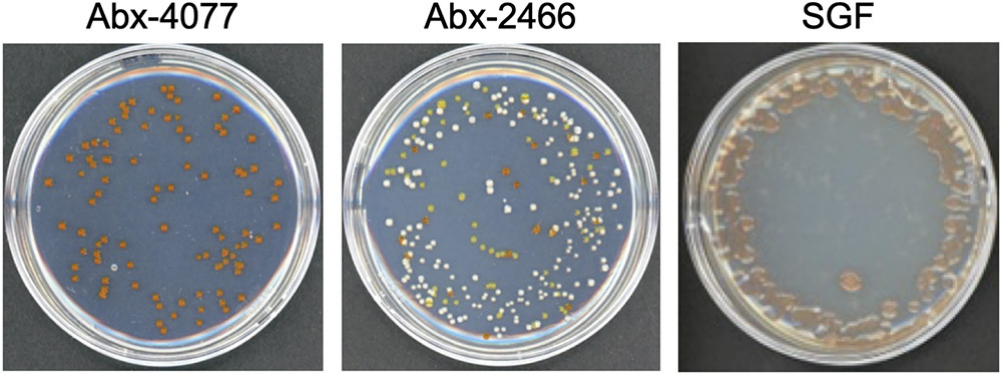
Colony-forming assays of bacteria in microalgal cultures after antibiotic treatment. Colony-forming assays were performed to evaluate bacterial survival after antibiotic treatment in F/2 agar medium. One thousand harvested cells of established cultures (Abx-4077, Abx-2466, and SGF) were resuspended in 100 μL of filtered seawater, and serial dilutions of samples were spread on marine agar plates without antibiotics. Numbers of colonies were counted after incubation at 25°C for 7 days.

The CFU counts per parent culture were 2.9 × 10^3^ and 4.5 × 10^4^ CFU/1,000 microalgal cells in NIES-4077 and CCMP2466 (Table 1). Of these, pigmented bacteria numbered 1.0 × 10^3^ and 2.4 × 10^4^ CFU, representing 36% and 53% of all bacterial CFU (Table 1). The CFU counts for the established cultures were 8.2 × 10^3^, 3.1 × 10^4^, and 2.2 × 10^2^ CFU/1,000 microalgal cells in Abx-4077, Abx-2466, and SGF, respectively (Fig. 1 and Table 1). Of these, pigmented bacteria numbered 8.2 × 10^3^ and 2.2 × 10^2^ CFU in Abx-4077 and SGF, respectively. Surprisingly, they represented 100% of all bacterial CFU in Abx-4077 and SGF; these results are consistent with those of our previous study, which found that a pigmented bacterium, GF1, was the dominant bacterial community after antibiotic treatment with F/2 agar medium containing KAS-antibiotics (25). In the Abx-2466 culture, pigmented bacteria were 7.3 × 10^3^ CFU, representing 23% of all bacterial CFU.

**TABLE 1:** Colony-forming assays of bacteria in microalgal cultures^*a*^

Parameter	NIES-4077	Abx-4077	CCMP2466	Abx-2466	SGF
CFU of total bacteria	2.9 ± 1.0 × 10^3^	8.2 ± 2.6 × 10^3^	4.5 ± 1.2 × 10^4^	3.1 ± 1.0 × 10^4^	2.2 ± 0.6 × 10^2^
CFU of pigmented bacteria	1.0 ± 0.3 × 10^3^	8.2 ± 2.6 × 10^3^	2.4 ± 0.9 × 10^4^	7.3 ± 1.4 × 10^3^	2.2 ± 0.6 × 10^2^
Pigmented bacteria (%)	36	100	53	23	100

a Each CFU value was the mean of three sample replicates. All data are given as means ± standard error of the mean.

Of the colonies grown on marine agar plates, we selected colonies showing orange pigmentation from Abx-4077 and Abx-2466, because they were commonly present in the bacterial communities of dinoflagellate cultures after antibiotic treatment (Fig. 1). Colonies showing orange pigmentation were purified with 1/10 strength ZoBell’s 2216E medium and were named bacterial strains C-4077 and C-2466, respectively. The 16S rRNA sequences of C-4077 and C-2466 showed 100% similarity to that of Maribacter flavus KCTC 42508 (30), which belongs to the family Flavobacteriaceae (Fig. S1). From the SGF culture, colonies showing pink pigmentation were purified and named bacterial strain DU1. The 16S rRNA sequence of DU1 showed 95.5% similarity to that of the most closely related strain, Roseivirga spongicola UST030701-084 (31), which belongs to the family Roseivirgaceae (Fig. S2).

### Bacterial community analysis of microalgal cultures before and after antibiotic treatment.

**FIG 2:**
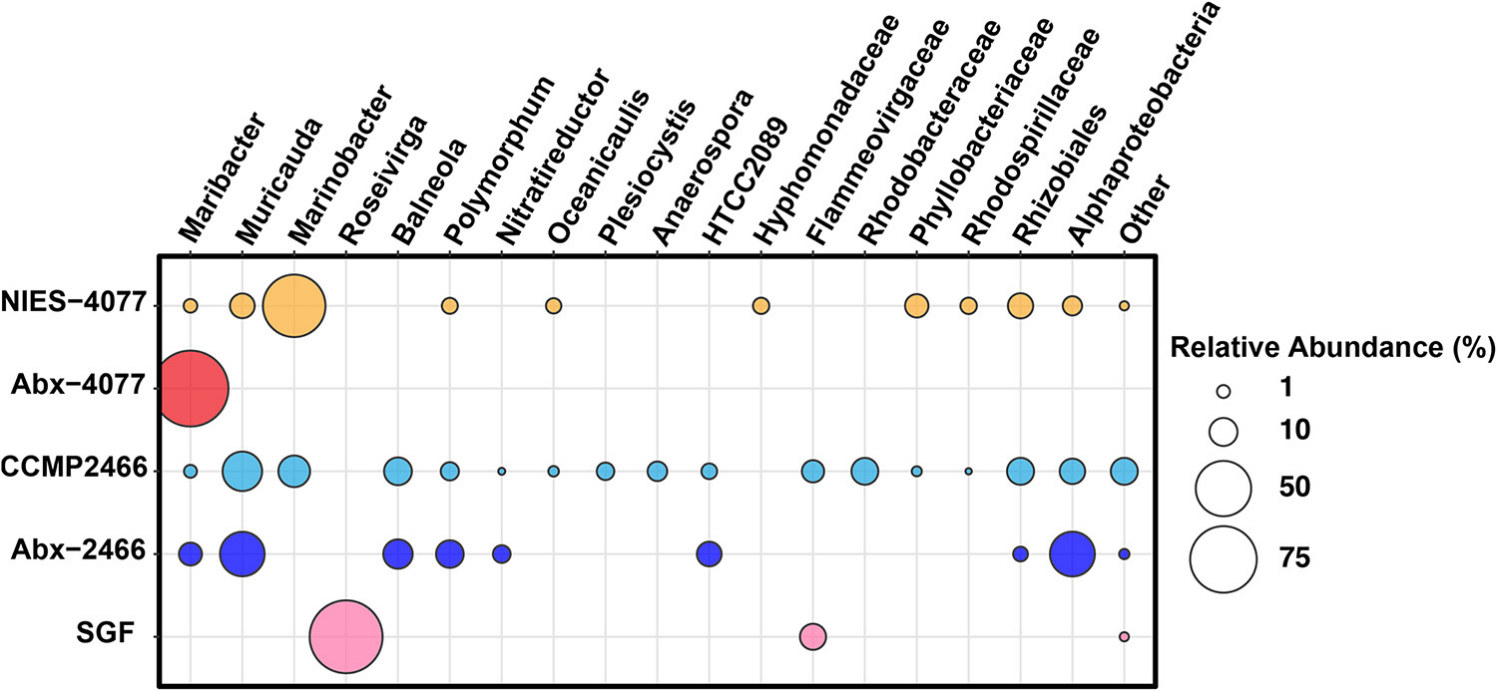
Bacterial community analysis of microalgal cultures before and after antibiotic treatment. Bacterial community composition (relative abundance %) of microalgal cultures before and after antibiotic treatment, based on extracted total DNA and 16S rRNA gene amplicon sequencing (Illumina MiSeq). Circle sizes represent relative abundances. Data are provided as means of relative abundances from three biological replicates. Abx-4077 and Abx-2466 were established on F/2 agar plates containing antibiotics (50 μg/mL kanamycin, 100 μg/mL ampicillin, and 50 μg/mL streptomycin) from NIES-4077 and CCMP2466, respectively. *Durusdinium* sp. strain SGF was isolated directly from the coral *Galaxea fascicularis* with F/2 agar plates supplemented with antibiotics. Bacterial taxa with >0.5% relative abundance in all three biological replicates in at least one microalgal culture were adopted.

To confirm changes in bacterial communities without a cultivation step, because some bacteria are unculturable on marine agar medium, we performed 16S rRNA amplicon sequencing of microalgal cultures before and after antibiotic treatment. Sequencing produced 912,197 reads (ranging from 47,327 to 86,899 reads per sample) across five Symbiodiniaceae cultures (*n *=* *3 per sample; 15 samples in total) (Table S1). After merging, denoising, and chimera filtering, 718,882 reads (ranging from 37,436 to 69,406 reads per sample) remained. Bacterial community composition before and after antibiotic treatment highlighted those differences (Fig. 2 and Table S2). The parent culture of NIES-4077 included sequences affiliated with *Maribacter*, *Muricauda*, *Marinobacter*, *Polymorphum*, and *Oceanicaulis* at the genus level and Hyphomonadaceae, Phyllobacteriaceae, and Rhodospirillaceae at the family level (Fig. 2). Sequences affiliated with *Marinobacter* were the most abundant, with a relative mean of 65.4% (ranging from 64.6% to 66.2% per sample) in the parent culture of NIES-4077. On the other hand, Abx-4077 was dominated by sequences affiliated with *Maribacter*, with a relative mean of 100%, which was consistent with the culture-dependent method (Fig. 1); while only having a relative mean abundance of 1.2% (ranging from 1.2% to 1.3% per sample) in the parent culture of NIES-4077. The parent culture of CCMP2466 included sequences affiliated with *Maribacter*, *Muricauda*, *Marinobacter*, *Balneola*, *Polymorphum*, *Nitratireductor*, *Oceanicaulis*, *Plesiocystis*, and *Anaerospora* at the genus level and Flammeovirgaceae, Rhodobacteraceae, Phyllobacteriaceae, Rhodospirillaceae, and HTCC2089 at the family level (Fig. 2). After antibiotic treatment, the dominant affiliated taxa shifted to *Maribacter*, *Muricauda*, *Balneola*, *Polymorphum*, and *Nitratireductor* at the genus level and HTCC2089 at the family level (Fig. 2) in Abx-2466. Among these taxa, *Muricauda* and *Maribacter* are pigmented bacteria, with a total of 36.4% of retrieved sequences (ranging from 33.7% to 38.6% per sample) in Abx-2466. The bacterial composition of SGF, freshly isolated from the coral with F/2 medium containing KAS-antibiotics, was dominated by sequences affiliated with *Roseivirga* at the genus level, with a relative mean of 91.5% (ranging from 88.5% to 93.3% per sample), and Flammeovirgaceae at the family level, with a relative mean of 8.3% (ranging from 6.6% to 11.1% per sample), which also agreed with the culture-dependent method (Fig. 1 and Table 1).

### Antibiotic-resistance of pigmented bacteria from the microalgal phycosphere.

**FIG 3:**
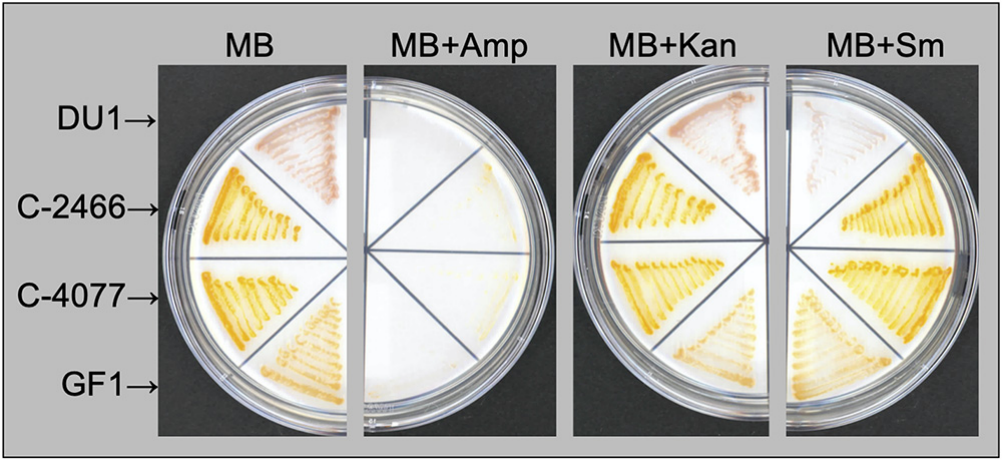
Antibiotic susceptibility tests of pigmented bacteria on marine agar plates. Pigmented strains (GF1, C-4077, C-2466, and DU1) were spread on marine agar (MB) plates with antibiotics (50 μg/mL of kanamycin [Kan], 100 μg/mL of ampicillin [Amp], or 50 μg/mL of streptomycin [Sm]) at 24°C for 4 to 6 days.

We confirmed that antibiotic treatment in F/2 agar medium (containing 50 μg/mL kanamycin, 100 μg/mL ampicillin, and 50 μg/mL streptomycin) altered bacteria community composition and selected mainly pigmented bacteria in Symbiodiniaceae cultures (Fig. 1 and Fig. 2). To further investigate interactions between Symbiodiniaceae and pigmented bacteria, we performed an antibiotic susceptibility test of Flavobacteriaceae sp. GF1 from the phycosphere of *Durusdinium* (25) and bacterial isolates from the present study (C-4077, C-2466, and DU1). All bacterial strains were able to grow on marine agar plates supplemented with 50 μg/mL kanamycin (Fig. 3). GF1, C-4077, and C-2466 were able to grow well on marine agar plates supplemented with 50 μg/mL streptomycin, but DU1 had low growth potential. In contrast, all bacterial isolates were incapable of growing on marine agar plates supplemented with 100 μg/mL ampicillin, indicating that these strains possess low resistance against ampicillin (Fig. 3).

Minimal inhibitory concentrations (MICs) and minimal bactericidal concentrations (MBCs) of kanamycin, ampicillin, and streptomycin were also obtained by broth microdilution assays of the four pigmented bacteria (GF1, C-4077, C-2466, and DU1) (Table 2). The MIC and MBC of Flavobacteriaceae sp. GF1 were 4 and 8 μg/mL of ampicillin, respectively. The MICs and MBCs of *Maribacter* spp. were between 8 and 32 μg/mL of ampicillin. In contrast, the MICs and MBCs for Flavobacteriaceae sp. GF1 and *Maribacter* spp. were more than the maximum concentrations (>64 μg/mL) of kanamycin and streptomycin, suggesting that the antibiotic concentrations in F/2 agar medium were insufficient to eliminate these strains. The MICs and MBCs of *Roseivirga* sp. DU1 were 16 and 32 μg/mL of ampicillin and streptomycin concentrations, whereas those concentrations of kanamycin were more than the maximum concentration (>64 μg/mL). Collectively, the concentrations in the F/2 agar medium used for antibiotic treatment (50 μg/mL kanamycin, 100 μg/mL ampicillin, and 50 μg/mL streptomycin) had sufficient bactericidal activity against all bacterial isolates.

**TABLE 2:** MICs and MBCs of antibiotics obtained by broth microdilution assays^*a*^

Strain	Antibiotic	MIC (μg/mL)	MBC (μg/mL)
Flavobacteriaceae sp. GF1	Amp	4	8
	Kan	>64	>64
	Sm	>64	>64
*Maribacter* sp. C-4077	Amp	8	16
	Kan	>64	>64
	Sm	>64	>64
Maribacter sp. C-2466	Amp	16	32
	Kan	>64	>64
	Sm	>64	>64
Roseivirga sp. DU1	Amp	16	32
	Kan	>64	>64
	Sm	32	32

a MIC, minimal inhibitory concentration; MBC, minimum bactericidal concentration; Amp, ampicillin; Kan, kanamycin; Sm, streptomycin.

### FISH analysis revealed pigmented bacteria on cell surfaces of dinoflagellates.

To reveal the localization of pigmented bacteria (C-4077 and C-2466 belonging to the Flavobacteriia, and DU1 belonging to the Cytophagia) in three Symbiodiniaceae cultures after antibiotic treatment (Abx-4077, Abx-2466, and SGF), we performed fluorescence *in situ* hybridization (FISH) using a cyamine 3 (Cy3)-labeled oligonucleotide probe, CF319a/b, to specifically target the 16S rRNA sequences of Flavobacteriia and Cytophagia (32). Before examining the localization of pigmented bacteria in the Symbiodiniaceae cultures, the hybridization of CF319a/b to pigmented bacteria (C-4077, C-2466, and DU1) was confirmed using bacterial cultures (Fig. S3).

**FIG 4:**
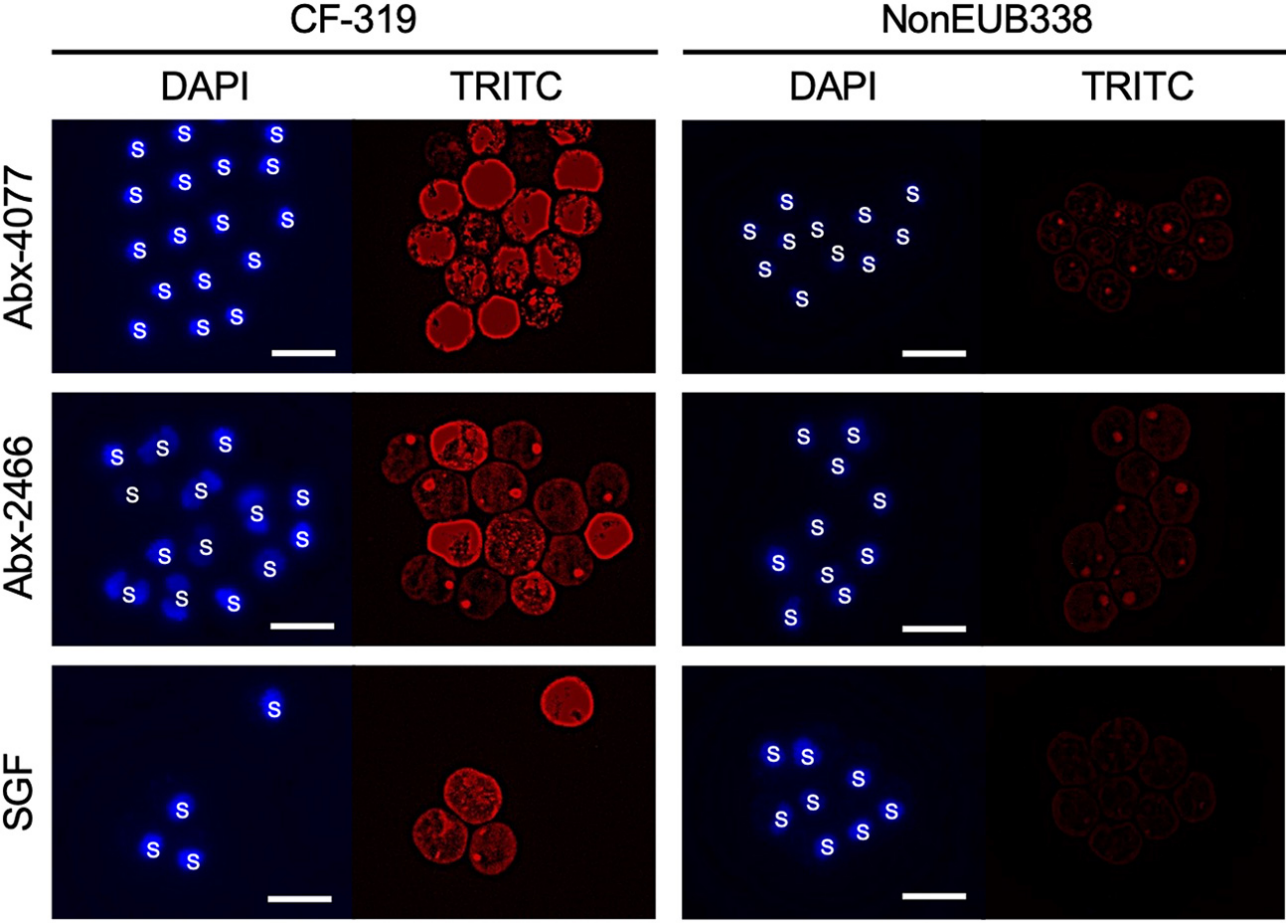
Fluorescence *in situ* hybridization (FISH) analysis of pigmented bacteria in microalgal cultures. DAPI (4′,6-diamidino-2-phenylindole) and cyamine 3 (Cy3) fluorescence were detected using a DAPI filter (excitation: 360/40 nm; emission: 460/50 nm; dichroic: 400 nm) and a TRITC (tetramethylrhodamine) filter (ex: 545/25 nm; em: 605/70 nm; dichroic: 565 nm), respectively. Microalgal samples were hybridized with CF319a/b (left panel) and NonEUB338 (right panel) probes labeled with Cy3. Scale bars = 10 μm. S, Symbiodiniaceae.

In microalgal cultures, Cy3 signals were detected from bacteria on the surface of Symbiodiniaceae cells from Abx-4077, Abx-2466, and SGF (Fig. 4), whereas no signal was detected in areas on the slide lacking Symbiodiniaceae cells (data not shown), indicating that these bacteria were not only present in the culture medium, but inhabited the cell surfaces of the microalgae. Although extended laboratory culture raises concerns about possibly artifactitious relationships between Symbiodiniaceae and pigmented bacteria, we found that DU1 was a symbiont of freshly isolated Symbiodiniaceae cells from the coral *G. fascicularis*. These results indicate that pigmented bacteria are closely associated with *in hospite* Symbiodiniaceae.

### *Maribacter* species produce zeaxanthin.

**FIG 5:**
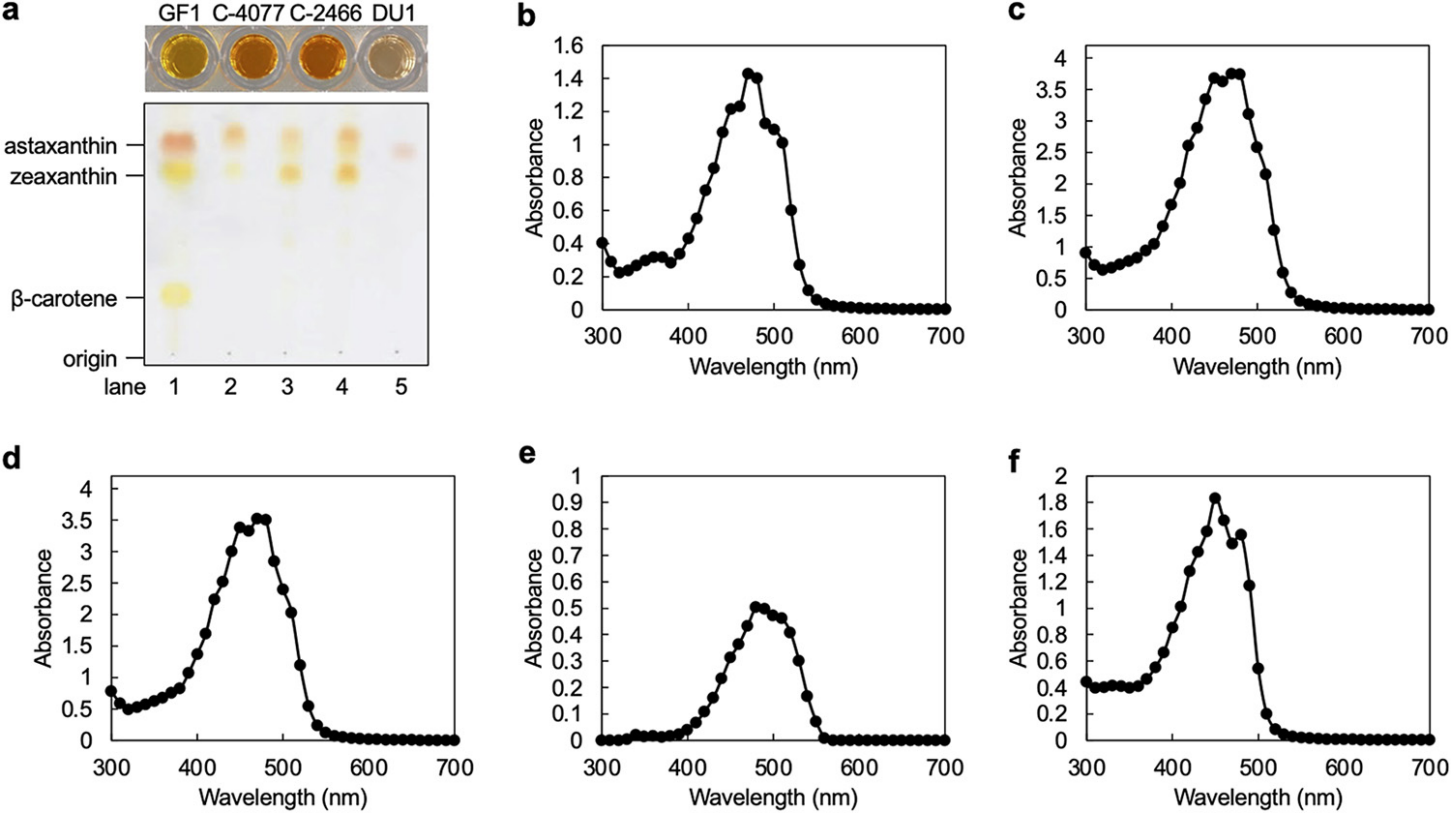
Analysis of carotenoids produced by bacterial isolates. (a) Extracts of pigmented bacteria and their TLC analyses. Cell pellets were extracted with acetone by vortexing. Reverse-phase thin-layer chromatography separation of synthetic carotenoid standards (lane 1) and pigments extracted from the pigmented bacteria listed below (lanes 2 to 5). Lane 2, GF1; lane 3, C-4077; lane 4, C-2466; lane 5, DU1. Absorption spectra of extracts of (b) GF1, (c) C-4077, (d) C-2466, (e) DU1, and (f) 0.1 mg/mL zeaxanthin standard.

The pigmented bacterium Flavobacteriaceae sp. GF1 was the dominant bacterial taxon after antibiotic treatment in F/2 agar medium containing KAS-antibiotics, and it protected its host Symbiodiniaceae from environmental stress through zeaxanthin production (25). Therefore, we performed reverse-phase thin-layer chromatography (TLC) analysis and a full-wavelength scan (300 to 700 nm) using a plate reader to identify carotenoids produced by bacterial isolates. Extractions of GF1, C-4077, and C-2466 showed an orange color, with C-4077 and C-2466 being a slightly darker orange (Fig. 5a). The extract of DU1 was pink (Fig. 5a), and the color disappeared when methanol was used as the extraction solvent (data not shown). Extractions of GF1, C-4077, and C-2466 were separated into two spots, indicating that these bacterial isolates at least zeaxanthin synthesized (Fig. 5a). The pink pigment could not be identified, but its mobility was intermediate between those of zeaxanthin and astaxanthin.

The absorption spectra of extracts were observed with full-wavelength scanning. The absorption peaks of extracts were 470 nm for GF1; 450 and 470 nm for C-4077; 450 and 470 nm for C-2466; 480 nm for DU1; and 450 and 480 nm for zeaxanthin standard (Fig. 5b to f). The maximum absorbance of zeaxanthin is 445 to 472 nm in dichloromethane and 451 nm in ethanol, which falls within the blue wavelength range (33–35). These results were consistent with the results of TLC analysis and absorption spectra of extracts (Fig. 5a to f). The pink pigment of DU1 showed weak absorption but, as in the TLC analysis, its spectrum did not permit identification.

### *Maribacter* affects stress tolerance of cultured Symbiodiniaceae.

**FIG 6:**
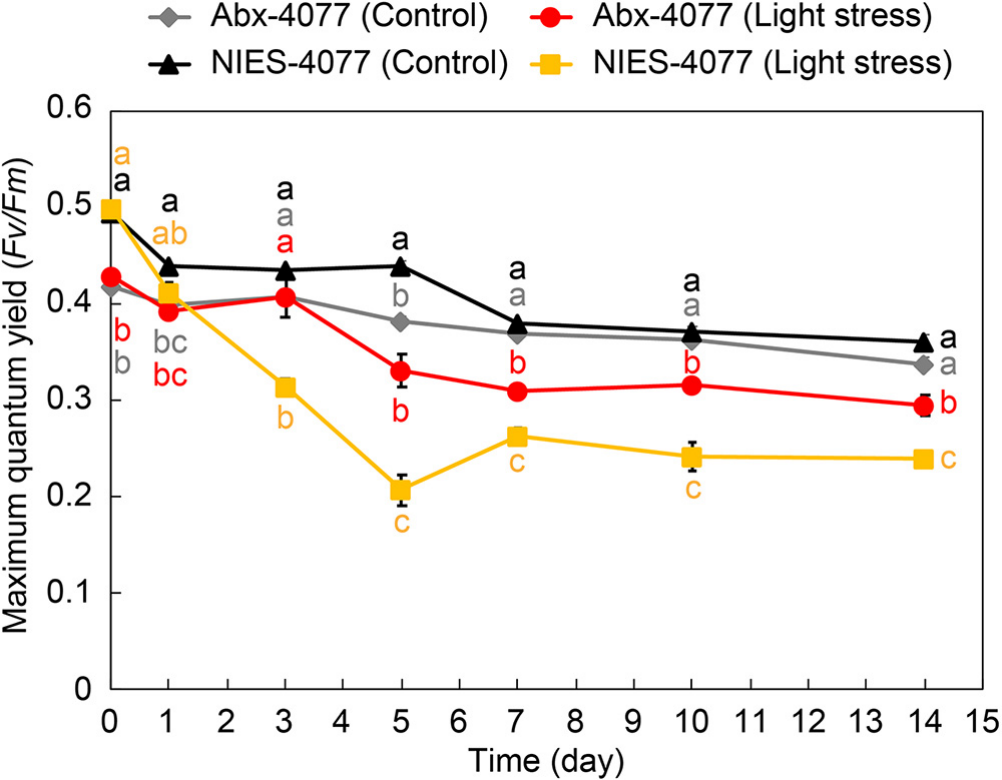
Effect of bacterial communities on the light tolerance of cultured Symbiodiniaceae. Maximum quantum yield of photosystem II (PSII) (variable fluorescence divided by maximum fluorescence [*Fv/Fm*]) in cultured Symbiodiniaceae under non-stressful lighting (24°C, 50 μmol photons m^−2^ s^−1^) and stressful lighting (24°C, 400 μmol photons m^−2^ s^−1^). To analyze the effects of light stress on dinoflagellate cells, we compared the *F_v_*/*F_m_* of NIES-4077 and Abx-4077 cells under stressful and non-stressful lighting at the same time. All data are given as means ± standard error of the mean (*n *=* *3). Statistical analysis was conducted using one-way analysis of variance followed by Tukey’s *post hoc* test for multiple-comparison tests. *P* values less than 0.05 were considered significant. Significant differences are represented by different letters. Colors of letters correspond to each sample (gray; Abx-4077 [Control], red; Abx-4077 [light stress], black; NIES-4077 [Control], yellow; NIES-4077 [light stress]). Abx-4077 was dominated by sequences affiliated with *Maribacter*, with a mean abundance of 100%, which had a relative mean abundance of 1.2% in the parent culture of NIES-4077.

*Maribacter* spp. inhabited the cell surfaces of dinoflagellate hosts and produced carotenoids, including zeaxanthin (Fig. 4 and Fig. 5), with maximal absorbance between 450 and 470 nm (Fig. 5c and d). In addition, pigmented bacteria increased from 1.0 × 10^3^ CFU in NIES-4077 to 8.2 × 10^3^ CFU in Abx-4077 (Table 1), and the relative mean abundances of sequences affiliated with *Maribacter* increased from 1.2% in NIES-4077 to 100% in Abx-4077 (Fig. 2). Next, we hypothesized that this microbiome manipulation with antibiotic treatment would affect the irradiation stress tolerance of cultured Symbiodiniaceae. To examine this hypothesis, we exposed microalgal cultures to irradiation stress for 14 days (at 24°C, light intensity of 400 μmol photons m^−2^ s^−1^). NIES-4077 showed a significantly higher maximum quantum yield of photosystem II (PSII) (variable fluorescence divided by maximum fluorescence [*F_v_*/*F_m_*]) on day 0 (prior to stress exposure) (Fig. 6). Conversely, Abx-4077 showed significantly higher *F_v_*/*F_m_* on day 3 after light exposure, which continued until day 14. These results indicated that *Maribacter* sp. C-4077 improved the irradiation stress tolerance of cultured Symbiodiniaceae.

## DISCUSSION

The relationships between Symbiodiniaceae hosts and the bacterial symbionts which protect them from environmental stress are important because coral holobionts are threatened by environmental stress associated with global warming (25). In a previous study, we isolated *Durusdinium* cells from the coral G. fascicularis using F/2 agar medium containing KAS-antibiotics (50 μg/mL kanamycin, 100 μg/mL ampicillin, and 50 μg/mL streptomycin) (25). The bacterial community of *Durusdinium* cells was dominated by orange-pigmented bacteria belonging to Flavobacteriaceae sp. GF1, which protected their microalgal hosts from heat and irradiation stress through zeaxanthin production. To confirm that KAS-antibiotic-induced selection of pigmented bacteria is a general phenomenon, we investigated the bacterial community changes of *Cladocopium* cells and *in hospite* Symbiodiniaceae from the coral *G. fascicularis* using colony-forming assays and 16S rRNA amplicon sequencing.

The results of colony-forming assays and 16S rRNA amplicon sequencing were consistent with those of our previous study (25), showing that only pigmented bacteria colonized marine agar plates in Abx-4077 and SGF (Fig. 1), and their relative mean abundances in the bacterial community were 100% and 91.5%, respectively (Fig. 2). Surprisingly, even though pigmented bacteria survived antibiotic treatment for 50 days in Symbiodiniaceae cultures, antibiotic concentrations in the F/2 agar medium had sufficient bactericidal activity against all bacterial isolates (Fig. 3 and Table 2). Since all surviving pigmented bacteria inhabited the cell surfaces of the Symbiodiniaceae hosts (Fig. 4), these results indicated that a close association with the microalgal cell wall conferred antibiotic resistance to the bacteria. Although both *Maribacter* and *Muricauda* were present in the NIES-4077 culture, only *Maribacter* remained viable after antibiotic treatment. On the other hand, both *Maribacter* and *Muricauda* survived in Abx-2466 culture, indicating that survival of bacteria under this antibiotic treatment varies depending on culture conditions, interactions with microalgae, and the duration of antibiotic treatment. Additionally, because the bacterial abundance of CCMP2466 was 15-fold higher than that of NIES-4077 based on colony-forming assays (Table 1), initial bacterial abundance may also be related to bacterial survival after antibiotic treatment. In addition, all pigmented bacteria acquired antibiotic resistance by close association with the microalgal cell wall, since these bacteria possess low resistance to ampicillin (MBCs ranged from 8 to 32 μg/mL) (Table 2). The depth to which bacteria embed in the Symbiodiniaceae cell wall may determine whether they survive antibiotic treatment. In other words, KAS-antibiotic treatment may select bacteria which have more intimate symbiotic relationships with Symbiodiniaceae and effectively invade the cell walls of their host microalgae. Hereafter, genome analysis of pigmented bacteria to search for antibiotic resistance genes and determine the mechanism of their close relationships with Symbiodiniaceae hosts is required.

Some microalgae have close physical and physiological relationships with symbiotic bacteria at the cell surface (36–38). Alavi et al. (36) found bacteria, including members of the *Roseobacter* clade and the alphaproteobacteria, which adhered to the cell surfaces of dinoflagellates. Loss of these bacteria from microalgal hosts significantly reduced the growth rate of dinoflagellates, which ultimately die without symbiotic bacteria. Pseudomonas spp. and *Rhizobium* spp. form biofilms with the green alga Botryococcus braunii that adhere to the surfaces of microalgal cells and secrete acyl-homoserine lactones (AHLs), involved in biofilm formation (37). AHLs are quorum-sensing-associated signaling molecules and regulate biofilm formation and virulence behaviors of bacteria (39). Colonial *B. braunii* cells extend fibrillar sheaths, mainly composed of polysaccharides, from the outer faces of their cell walls into the growth medium (40). Because NIES-4077 and CCMP2466 also formed colonies on F/2 agar medium containing KAS-antibiotics and secreted an extracellular matrix (ECM) around the periphery of these colonies (Fig. S4), the ECM involving biofilm formation produced by the bacteria themselves or their microalgal hosts may provide antibiotic resistance to pigmented bacterial symbionts.

These studies suggest that some macroalgae and bacteria form strong symbiotic relationships (41). Five bacterial genera (*Maribacter*, *Muricauda*, *Marinobacter*, *Polymorphum*, and *Oceanicaulis*) were commonly present in the parent cultures, NIES-4077 and CCMP2466. Among these, *Muricauda* and *Marinobacter* have been reported as core members of other Symbiodiniaceae cultures (27). The carotenoid-producing Flavobacteriaceae sp. strain GF1, which is most closely related to the genus *Muricauda*, has been isolated from the phycosphere of *Durusdinium* (25). Antibiotic treatment eliminated GF1 from cultured *Durusdinium*, resulting in a decreased *F_v_*/*F_m_* and increased ROS production under thermal and light stresses. GF1 inoculation in antibiotic-treated *Durusdinium* cultures restored the *F_v_*/*F_m_* and reduced ROS generation. Therefore, it is likely that *Muricauda* also protects microalgal hosts in NIES-4077 and CCMP2466. *Marinobacter* produces siderophores which increase iron bioavailability to phytoplankton (24) and this may have a positive impact on the growth of Symbiodiniaceae cells (42). Surprisingly, *Marinobacter* had the highest relative abundance based on 16S rRNA amplicon sequencing in NIES-4077, accounting for 65.4%, but 0% after antibiotic treatment (Fig. 2 and Table S2). Conversely, *Maribacter* accounted for 1.2%, but 100% after antibiotic treatment, indicating that *Maribacter* has a strong symbiotic relationship with NIES-4077. Furthermore, this microbiome manipulation of the bacterial community reduced the *F_v_*/*F_m_* under non-stress conditions (Fig. 6), suggests that members of the bacterial community, such as *Muricauda* and *Marinobacter*, which were eliminated by the antibiotic treatment, are directly or indirectly involved in photosynthesis by dinoflagellates in their natural environment, e.g., via siderophore or carotenoid production.

On the other hand, *Maribacter* produced carotenoids, including zeaxanthin, which have a maximal absorbance between 450 and 470 nm (Fig. 5), and enhanced the maintenance function of *F_v_*/*F_m_* under irradiation stress, suggesting that they mitigate environmental stress by producing carotenoids on the cell walls of Symbiodiniaceae. The details of this mechanism are not known, but zeaxanthin produced by *Maribacter* may function as a “sunscreen” on dinoflagellate cells due to its photoprotective function. Furthermore, zeaxanthin, a xanthophyll carotenoid produced by photosynthetic organisms such as plants and algae (43, 44), accumulates via the xanthophyll cycle in response to excess solar irradiation and induces non-photochemical quenching (NPQ) by dissipating excess excitation energy as heat (45). Because xanthophyll cycle pigments comprise diadinoxanthin and diatoxanthin (which is functionally equivalent to zeaxanthin) in microalgae such as dinoflagellates and diatoms (46, 47), zeaxanthin produced by *Maribacter* could be converted into diatoxanthin in dinoflagellates to induce NPQ, thereby protecting the microalgal host from light stress. Future studies should evaluate the dynamics of zeaxanthin in the phycosphere to identify the mechanisms by which pigmented bacteria protect cultured dinoflagellates from environmental stresses.

*Cladocopium* strains (NIES-4077 and CCMP2466) were originally isolated from the bivalve *Fragum* sp. in Okinawa, Japan (29) and from the anemone Discosoma sanctithomae in the Caribbean Sea (https://ncma.bigelow.org/ccmp2466). We propose that given its conserved occurrence in two strains 10,000 km apart, it is possible that *Cladocopium* is closely associated with bacteria of the genus *Maribacter*. Although the detailed functions of this bacterium in association with microalgae are largely unknown, *Maribacter* is often isolated from seawater, marine sediments, and macroalgae (48–53). In macroalgae, the genus *Maribacter* induces morphogenesis by producing various stimulatory chemical mediators (52, 53), so it may function similarly in symbiotic dinoflagellates, not only in photoprotection via carotenoid production but also in promoting the growth of the host microalgae. The mechanism by which pigmented bacteria such as *Maribacter*, *Muricauda*, and *Roseiviriga* maintain such strong symbiotic relationships with Symbiodiniaceae remains an open question, but one hypothesis is that symbiotic relationships between Symbiodiniaceae and pigmented bacteria may have been positively selected because the bacteria mitigate environmental stresses, such as excess insolation and higher temperatures, through carotenoid production on microalgae cell walls.

In conclusion, we demonstrated that KAS-antibiotics select pigmented bacteria in cultures of coral-symbiotic dinoflagellates and that these bacteria form strong associations with the cell walls of their host microalgae. These results demonstrate that bacteria of low abundance under “natural” conditions, i.e., no antibiotic treatment, have potentially beneficial functions in cultures of endosymbiotic dinoflagellates. The present study provides important new insights into dinoflagellate-bacteria interactions. Dinoflagellate cells protected the bacteria from antibiotics, while bacteria protected microalgal cells from light stress via carotenoid production. Furthermore, microbiome manipulation with KAS-antibiotics maintained higher photosynthetic activity under light stress compared to that in the parental strain. To the best of our knowledge, this is the first report of improved stress tolerance of Symbiodiniaceae over parental strains by microbiome manipulation. Although coral probiotics have been investigated in several studies (16–19), ROS generation from symbiotic microalgae induced by long-term stress due to high temperatures and excess light is a major cause of the breakdown of symbiosis between host coral animals and unicellular, photosynthetic dinoflagellates. Future studies should investigate the beneficial bacteria which inhabit Symbiodiniaceae cell surfaces and can scavenge ROS as a potential solution to mass bleaching events. Corals reacquire microalgal partners from marine environment, so creating multiple stress-tolerant Symbiodiniaceae cells, i.e., heat and irradiation stress-tolerant, through bacterial manipulation and the reconstruction of symbiotic relationships between endosymbiotic dinoflagellates and corals for microbiome manipulation of the coral holobiont may prove useful for coral reef conservation.

## MATERIALS AND METHODS

### Microalgal cultures and growth conditions.

*Cladocopium* sp. (clade C strain, ID: NIES-4077), originally isolated from the bivalve *Fragum* spp., was provided by NIES through the National Bio-Resource Project (NBRP) of the MEXT (Japan) (29). Cladocopium goreaui (clade C1 strain, ID: CCMP2466), originally isolated from the anemone Discosoma sanctithomae was obtained from the Bigelow Laboratory for Ocean Sciences (West Boothbay Harbor, ME, USA; https://ncma.bigelow.org/ccmp2466). Dinoflagellate cells were grown in F/2 liquid medium (G0154, Sigma-Aldrich, St. Louis, MO, USA) without antibiotics at 24°C and a light intensity of 50 μmol photons m^−2^ s^−1^ on a 12:12 h light:dark cycle. A light meter (LI-250A; LI-COR Biosciences, Lincoln, NE, USA) was used to measure light intensity.

### Antibiotic treatment of Symbiodiniaceae species.

Antibiotic treatment of Symbiodiniaceae species was performed as previously described (25). Briefly, Symbiodiniaceae cells in culture were counted with a Neubauer-improved counting chamber under a microscope (Leica DM2000 LED, Leica, Wetzlar, Germany), and approximately 10^3^ cells were harvested by centrifugation at 800 × *g* for 5 min at 25°C. Collected cells were resuspended in 100 μL F/2 medium and incubated on F/2 agar plates supplemented with KAS-antibiotics (50 μg/mL kanamycin, 100 μg/mL ampicillin, and 50 μg/ of streptomycin) at 24°C and a light intensity of 50 μmol photons m^−2^ s^−1^ on a 12:12 h light:dark cycle for 50 days. Colonies were picked and incubated in 5 mL F/2 liquid medium without antibiotic under the conditions described above and subcultured every 30 to 50 days until the start of experimental trials. The resulting strains were named Abx-4077 and Abx-2466, respectively.

### Culture of coral *G. fascicularis*.

Colonies of *G. fascicularis* were collected around Sesoko Island, Okinawa, on 7 December 2020 and transported to the laboratory at the University of Tokyo (Kashiwa, Chiba Prefecture). Corals were kept at 26°C in an aquarium containing 600 L of artificial seawater prepared with Viesalt (Marine Tech, Tokyo, Japan), and 40% of the seawater was replaced every 2 weeks. The aquarium was equipped with four light-emitting diode (LED) lamps (Radion XR15w G4 Pro light; EcoTech Marine, Allentown, PA, USA), a protein skimmer (ReefLive Inspire2200; LSS laboratory, Tokyo, Japan), and a calcium reactor (ReefLive VCAL1200; LSS laboratory). Corals were fed three times per week with frozen copepods (Clean Copepoda; Kyorin Co., Hyogo, Japan), frozen mysids (Clean White Shrimp; Kyorin Co.), and frozen adult *Artemia* (Clean Brine Shrimp; Kyorin Co.). Salinity was tested daily with a Marine Salinity Tester (cat no. HI98319; Hanna Instruments, Woonsocket, RI, USA), and adjusted to 1.025 SG. Phosphate was measured using a Phosphorus Marine Ultra-Low Range Colorimeter (HI736; Hanna Instruments) and maintained between 5 and 20 ppb. Alkalinity was tested using a Seawater/Marine Alkalinity (dKH) Colorimeter (HI772; Hanna Instruments) and kept between 6.0 and 8.0 dKH. Permits for coral collection were obtained from the Okinawa Prefectural Government for research use (permit no. 2-46).

### Fresh isolation and cultivation of symbiotic dinoflagellates from corals.

Isolation of endosymbiotic dinoflagellates was performed according to the method described by Motone et al. (25). In brief, a *G. fascicularis* colony was fragmented to obtain single polyps and centrifuged at 100 × *g* for 10 s in a 1.5-mL tube to remove surface-associated seawater. Polyp tissue was harvested by further centrifugation at 8,000 × *g* for 2 min, after which the pellet was suspended in 1 mL of F/2 liquid medium. Next, the tissue suspension was serially diluted and incubated on F/2 agar plates supplemented with KAS-antibiotics (50 μg/mL kanamycin, 100 μg/mL ampicillin, and 50 μg/mL streptomycin) at 24°C and a light intensity of 50 μmol photons m^−2^ s^−1^ on a 12:12 h light:dark cycle for 50 days in a cool incubator (CN-25C, Mitsubishi Electric Co., Tokyo, Japan) with LED lamps using blue LED chips peaked at around 450 nm and yellow-emitting phosphor peaked at around 550 nm (Clear LED Power X 2030; GEX Corporation, Osaka, Japan). Colonies were subsequently incubated in F/2 liquid medium without antibiotics under the conditions described above and subcultured every 30 to 50 days until the start of experimental trials. Isolated dinoflagellates were identified as *Durusdinium* (formerly *Symbiodinium* clade D) (8), based on ITS2 region sequences (54), and were named strain SGF.

### Colony-forming assays and isolation of bacteria from endosymbiotic dinoflagellates.

Colony-forming assays were performed to evaluate bacterial abundance before and after antibiotic treatment in F/2 agar medium. Approximately 10^3^ harvested cells from microalgal cultures before and after antibiotic treatment were resuspended in 100 μL of filtered seawater, and serially diluted samples were spread onto marine agar plates without antibiotics (BD Biosciences, Franklin Lakes, NJ, USA). Symbiodiniaceae cells in culture were counted with a Neubauer-improved counting chamber under a microscope (Leica DM2000 LED). Numbers of colonies were counted after incubation at 25°C for 7 days.

Colonies showing orange pigmentation were isolated from *Cladocopium* sp. strain NIES-4077 and *C. goreaui* strain CCMP2466. Bacterial colonies were purified in 1/10 strength ZoBell’s 2216E agar medium (0.05% [wt/vol] peptone, 0.01% [wt/vol] yeast extract, 1.5% [wt/vol] agar in 1 L filtered seawater), and named bacterial strains C-4077 and C-2466, respectively. Colonies showing pink pigmentation were also isolated from *Durusdinium* sp. strain SGF. Bacterial colonies were purified with 1/10 strength ZoBell’s 2216E medium and named bacterial strain DU1.

### Phylogenetic analysis of C-4077, C-2466, and DU1.

Genomic DNA was isolated from bacterial strains C-4077, C-2466, and DU1 grown in marine broth (BD Biosciences) using the Qiagen Genomic-tip 20/G and Qiagen DNA Buffer Set (Qiagen, Hilden, Germany). The 16S rRNA genes of these strains were PCR-amplified using KOD FX Neo (TOYOBO, Osaka, Japan) and the primers 8F (5′-AGAGTTTGATCMTGGCTCAG-3′) and 1492R (5′-GGTTACCTTGTTACGACTT-3′), according to the manufacturer’s instructions. PCR cycling conditions were as follows: initial denaturing at 94°C for 2 min; 30 cycles of 98°C for 10 s, 53°C for 30 s, and 68°C for 50 s; and a final extension at 68°C for 3 min. The 16S rRNA genes of C-4077, C-2466, and DU1 were sequenced (Eurofins Genomics, Ebersberg, Germany) and deposited in the DDBJ/EMBL/GenBank databases under the accession numbers LC699426, LC699427, and LC699428. The 16S rRNA gene sequences of C-4077, C-2466, and DU1 were compared with those of the type strains of closely related species using the Nucleotide Similarity Search program (EzBioCloud, www.ezbiocloud.net/) (55). A phylogenetic tree was constructed based on the 16S rRNA sequences using the neighbor-joining method (1,000 bootstrap replicates) using MEGA11 (56), after multiple data alignments with the online version of MAFFT v7 (https://mafft.cbrc.jp/alignment/software/) (57). Distances were calculated with the Kimura two-parameter model for DNA analysis (58).

### 16S rRNA amplicon sequencing and data analysis.

Dinoflagellate cells (5.0 × 10^5^ to 1.0 × 10^6^) were harvested via centrifugation at 3,000 × *g* for 5 min and immediately frozen in liquid nitrogen. DNA from frozen cells was extracted using a PowerSoil Pro DNA isolation kit (Qiagen) according to the manufacturer’s instructions. Paired-end (2 × 300 bp) DNA sequencing of the 16S rRNA V1/V2 region was performed on an Illumina MiSeq platform (Illumina, San Diego, CA, USA) with the MiSeq reagent kit v3 (Illumina) by Bioengineering Lab. Co., Ltd. (Kanagawa, Japan), as previously described (16). The variable region V1/V2 of the 16S rRNA gene was amplified using 27Fmod-338R primers (59). The first PCR amplifications were performed in a final volume of 25 μL containing 12.5 μL of KAPA HiFi HotStart ReadyMix, 1 μL of each primer (10 μM), and 5 ng of template DNA. PCR cycling conditions were as follows: initial denaturing at 94°C for 2 min; 25 cycles of 94°C for 30 s, 50°C for 30 s, and 72°C for 30 s; and a final extension at 72°C for 5 min. The second PCR using TaKaRa *Ex Taq* HS DNA polymerase (TaKaRa-Bio, Kusatsu, Japan) was performed according to the following protocol: initial denaturing at 94°C for 2 min; 12 cycles of 94°C for 30 s, 60°C for 30 s, and 72°C for 30 s; and then a final extension at 72°C for 5 min. Amplified DNA was purified using Agencourt AMPure XP (Beckman Coulter, Inc., Brea, CA, USA) after each amplification step.

Quality filtering was performed using the FASTX-Toolkit v0.0.14 (60) and sickle v1.33 (61) (with a Q-score of <20 and a length of <130). Paired sequence reads were assembled using FLASH v1.2.11 (62) with a minimum overlap of 10 bp. QIIME2 (v2021.4), with default parameter values, was used for sequence denoising using the DADA2 plugin for chimera checking (63, 64). Surviving sequences were then clustered into amplicon sequence variant taxonomic classifications with 97% similarity and classified using the Greengenes database (v13_8) (65). Bacterial taxa with more than 0.5% relative abundance which were present in all three biological replicates in at least one microalgal culture were adopted.

### Antibiotic susceptibility test on bacterial isolates.

To confirm whether isolates could grow in medium containing antibiotics, Flavobacteriaceae sp. strain GF1 (25) and the pigmented strains isolated in the present study (C-4077, C-2466, and DU1) were spread on marine agar plates (BD Biosciences) with antibiotics (50 μg/mL kanamycin, 100 μg/mL ampicillin, or 50 μg/mL streptomycin) at 24°C for 4 to 6 days. Furthermore, to evaluate resistance against single antibiotics (kanamycin, ampicillin, or streptomycin), antibacterial susceptibility was assessed using a broth microdilution assay based on published procedures (66, 67). Bacterial strains were stored as frozen glycerol stock cultures at −80°C until use. All bacterial strains were cultivated on marine agar plates at 25°C and used for tests within 7 days. Twenty mg of cell pellets was harvested from agar plates using a sterile platinum loop and resuspended in 1 mL of marine broth. Each antibiotic was 2-fold serially diluted with a final concentration of 64 μg/mL to 1 μg/mL. Twenty-μL aliquots of antibiotics were incubated on microtiter plates with 100 μL of marine broth of each bacterial strain with 10^5^ CFU/mL. Antibacterial susceptibility was assessed by measuring bacterial growth after incubation with gentle shaking at 25°C for 72 to 96 h. Bacterial growth was monitored by measurements of the optical density at 600 nm (OD_600_) (SpectraMax iD3, Molecular Devices,LLC, San Jose, CA, USA). MICs were recorded as the lowest dilution inhibiting bacterial growth (measured at OD_600_) after incubation. MBCs were determined by plating the contents of the first three wells which had no visible bacterial growth onto marine agar plates and incubation at 25°C for 72 h. The lowest concentration of antibiotics that prevented colony formation was recorded as the MBC.

### Fluorescence *in situ* hybridization analysis of pigmented bacteria in microalgal cultures.

Bacterial and microalgal samples were fixed in 4% paraformaldehyde for 3 h (Wako, Osaka, Japan). Bacterial strains were directly collected from marine agar medium. After being washed with PBS (pH 7.4), samples were spotted onto APS-coated microscope slides (Matsunami Glass, Osaka, Japan), and air-dried, followed by serial dehydration in 50%, 80%, and 99.5% ethanol. Samples were hybridized with a probe targeting the 16S rRNA of Flavobacteriaceae (C-4077 and C-2466) and Cytophagia (DU1) bacteria (CF319a/b: 5′-TGGTCCGTRTCTCAGTAC-3′) and a nonsense, negative-control probe (NonEUB338: 5′-ACATCCTACGGGAGG-3′) (32, 68) labeled with Cy3 fluorochrome (Eurofins Genomics) in a hybridization buffer (formamide [35% for CF319a/b; 25% for NonEUB338], 0.9 M NaCl, 20 mM Tris/HCl, and 0.01% SDS) at 46°C for 2 h. After being washed with a washing buffer (0.08 M NaCl, 20 mM Tris/HCl, 5 mM EDTA, and 0.01% SDS) for 15 min, samples were counterstained with DAPI (4′,6-diamidino-2-phenylindole) in VECTASHIELD mounting medium (Vector Laboratories, CA, USA) and observed under an all-in-one fluorescence microscope (BZ-X800, Keyence, Osaka, Japan) equipped with an optical sectioning module (BZ-H4XF, Keyence). The optical sectioning module was only used to observe microalgal samples. Fluorescent signals were detected using a TRITC (tetramethylrhodamine) filter (excitation: 545/25 nm; emission: 605/70 nm; dichroic: 565 nm; OP-87764, Keyence) and a DAPI filter (ex: 360/40 nm, em: 460/50 nm, dichroic: 400 nm, OP-87762, Keyence) using Haze Reduction application (Keyence).

### Pigmentation analysis of bacterial isolates.

Pigmented strains (GF1, C-4077, C-2466, and DU1) were grown in marine broth for 48 h at 25°C and collected by centrifugation for 5 min at 3,000 × *g* at 25°C. Thirty mg (wet weight) of bacteria was then transferred to 100 mL of marine broth and further incubated for 48 h at 25°C. Prior to use in carotenoid extraction, the broth was centrifuged at 8,000 × *g* for 10 min at 4°C. The supernatant was removed, and cell pellets were washed twice with PBS. Cell pellets were extracted with acetone or ethanol using a vortex mixer for at least 30 min, and then cell bleaching was confirmed. For thin-layer chromatography, acetone extracts were spotted onto reverse-phase TLC plates (Silica-gel 60 RP-18 F_254_s; Merck, Darmstadt, Germany), and pigments were separated using a mobile phase of acetone/methanol/water (50/50/4 by volume). Ethanol extracts were used for full wavelength scans because the absorption peaks of acetone extracts were shifted (data not shown). Because the absorbance of an ethanol extract from DU1 was very low (Fig. S5), the absorption spectrum of an acetone extract is shown in Fig. 5e. Ethanol extracts were further diluted with ethanol and subjected to full-wavelength scans (300 to 700 nm) using a SpectraMax iD3 plate reader (Molecular Devices). A β-carotene standard was purchased from FUJIFILM Wako Pure Chemical Corporation (031-05533; Osaka, Japan). Astaxanthin and zeaxanthin standards were purchased from AK Scientific (V0395 and Q444; Union, CA, USA).

### Stress challenges and physiological assessment.

Dinoflagellate cells were acclimated in F/2 medium (*n *=* *3) in a tube (Eiken Centrifuge Tube; Eiken Chemical Co., Ltd., Tokyo, Japan) under the same culture conditions as described above for at least 3 days before stress exposure. For the light stress experiment, Symbiodiniaceae cells were incubated at 24°C under light (400 μmol photons m^−2^ s^−1^) and non-light stress (50 μmol photons m^−2^ s^−1^) for 14 days. *F_v_*/*F_m_* was measured from the bottoms of the tubes using a pulse-amplitude-modulated fluorometer (Junior-PAM; Walz, Effeltrich, Germany) after algal cultures had been dark-adapted for 30 min. The settings for junior PAM were as follows: measuring light = 9, saturation light = 8, and gain = 1.

### Statistical analysis.

To analyze effects of light stress on dinoflagellate cells, we compared the maximum quantum yield of PSII (*F_v_*/*F_m_*) of NIES-4077 and Abx-4077 cells under stressful and non-stressful lighting. All data are given as means ± standard error of the mean (*n *=* *3). Statistical analysis was conducted using one-way analysis of variance (ANOVA) followed by Tukey’s *post hoc* test for multiple-comparison tests in EZR software (Jichi Medical University Saitama Medical Center, Saitama, Japan; http://www.jichi.ac.jp/saitama-sct/SaitamaHP.files/statmedEN.html) (69), which is a graphical user interface for R (R Foundation for Statistical Computing, Vienna, Austria, v2.13.0). *P* values less than 0.05 were considered statistically significant.

### Data availability.

Raw sequencing data obtained via next-generation sequencing analysis are available in the DDBJ Sequence Read Archive under accession no. DRA013944.
