# Supplementary material for: Mutualistic Interactions between Dinoflagellates and Pigmented Bacteria Mitigate Environmental Stress
Source: Microbiol Spectr. 2023 Jan 18;11(1):e02464-22. doi: 10.1128/spectrum.02464-22 (PMC9927270; doi:10.1128/spectrum.02464-22)
Supplement: Supplemental file 1 — Fig. S1 to S5. Download spectrum.02464-22-s0001.pdf, PDF file, 2.7 MB [file spectrum.02464-22-s0001.pdf]

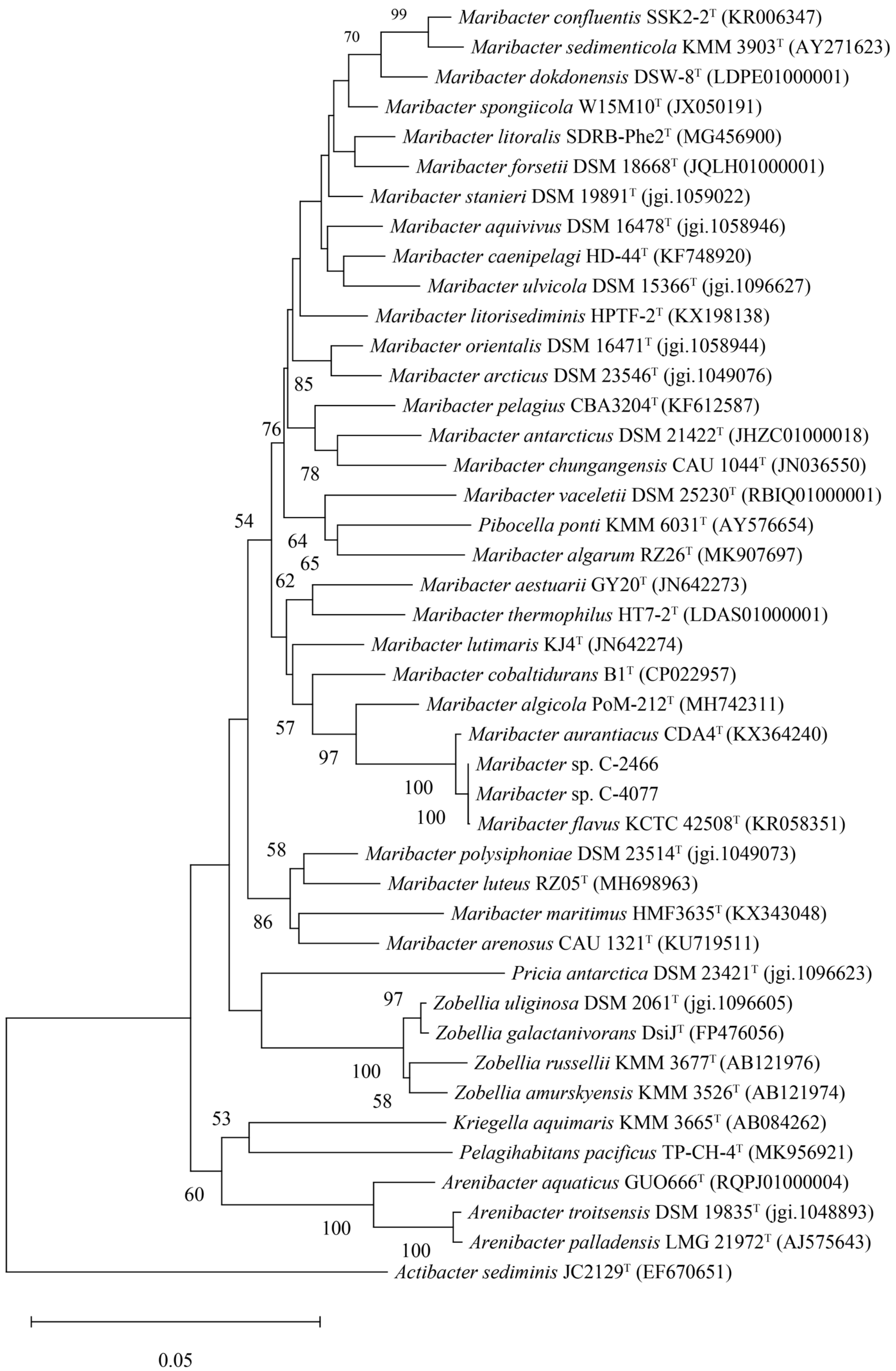

Figure S1. Neighbor-joining tree based on 16S rRNA gene sequences showing phylogenetic relationships of C-4077 and C-2466, and related taxa in the family Flavobacteriaceae. Numbers on branches represent bootstrap values (1,000 replications). Bootstrap values >50% are indicated at nodes. Bar, 0.05 substitutions per nucleotide position. The sequence of *Actibacter sediminis* JC2129^{T} was used as the outgroup.

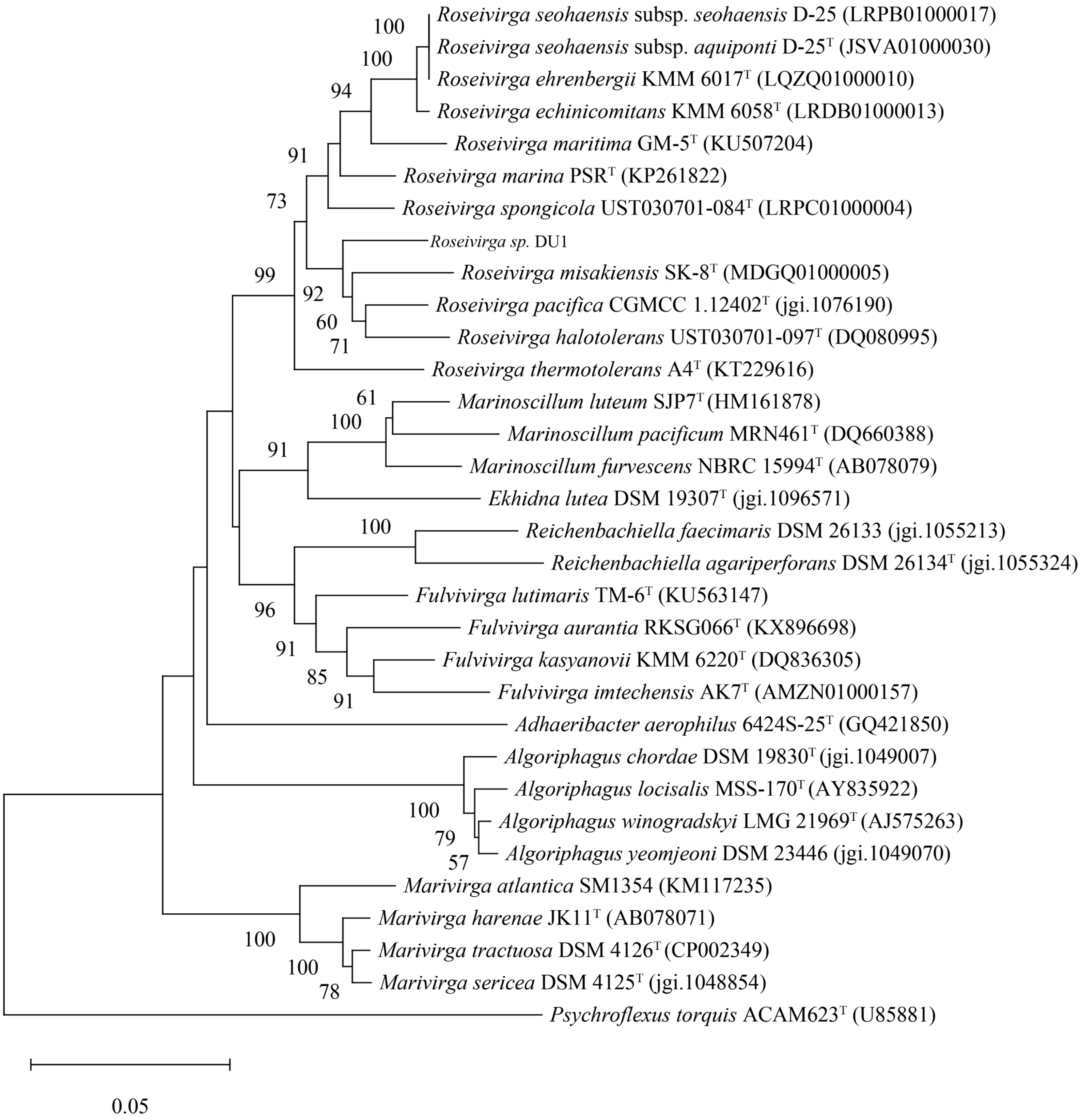

Figure S2. Neighbor-joining tree based on 16S rRNA gene sequences showing phylogenetic relationships of DU1 and related taxa in the family Roseivirgaceae.

Numbers on branches represent bootstrap values (1,000 replications). Bootstrap values >50% are indicated at nodes. Bar, 0.05 substitutions per nucleotide position. The sequence of *Psychroflexus torquis* ACAM623^T was used as the outgroup.

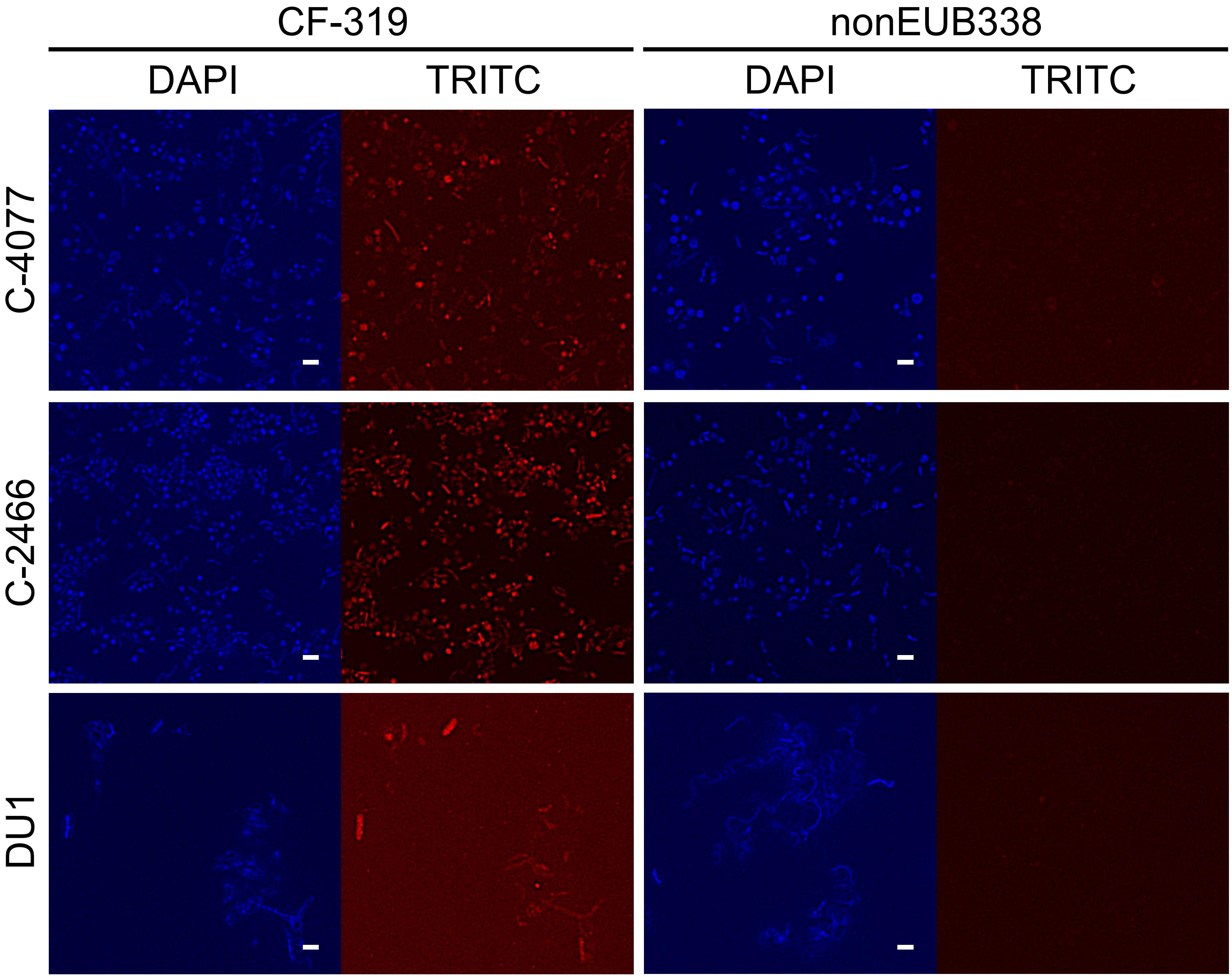

Figure S3. Fluorescence *in situ* hybridization (FISH) analysis of pigmented bacteria.
DAPI and Cy3 fluorescence were detected using a DAPI filter (ex: 360/40 nm, em: 460/50 nm, dichroic: 400 nm) or a TRITC filter (ex: 545/25 nm, em: 605/70 nm, dichroic: 565 nm), respectively. Bacterial samples were hybridized with CF319a/b (left panel) and NonEUB338 (right panel) probes labeled with Cy3. Scale bars are 2 μm.

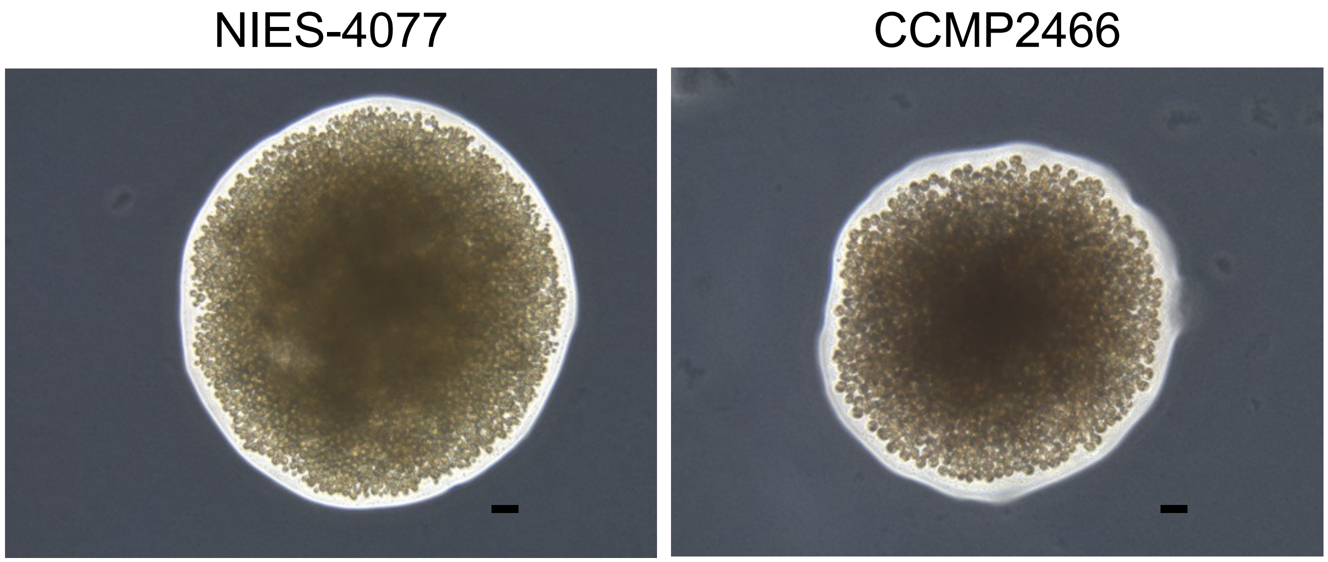

Figure S4. Colonies of NIES-4077 and CCMP2466 on f/2 agar medium containing KAS-antibiotics

NIES-4077 and CCMP2466 formed colonies on f/2 agar medium containing KAS-antibiotics and secreted an extracellular matrix (ECM) around the periphery of colonies. Colonies were observed using an all-in-one fluorescence microscope (BZ-X800, KEYENCE, Osaka, Japan). The scale bar is 30 μm.

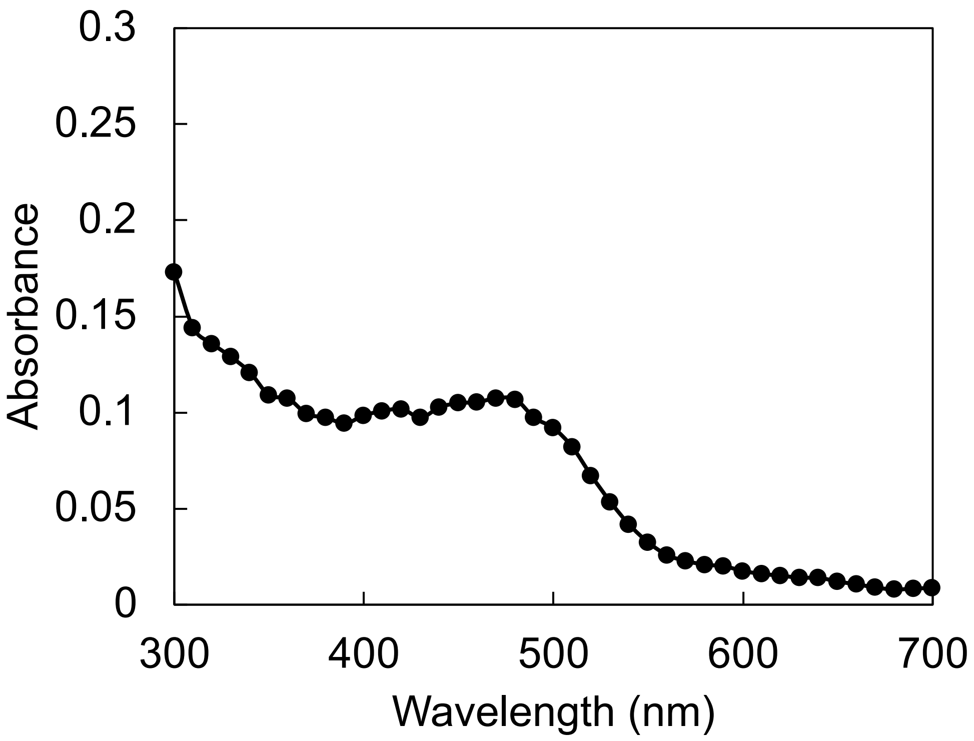

Figure S5. Absorption spectrum of an ethanol extract of DU1
